# Supplementary material for: HIVprotI: an integrated web based platform for prediction and design of HIV proteins inhibitors
Source: J Cheminform. 2018 Mar 9;10:12. doi: 10.1186/s13321-018-0266-y (PMC5845081; doi:10.1186/s13321-018-0266-y)
Supplement: Supplementary file 2 — Additional file 2. Source code of HIVProtI web server. [file 13321_2018_266_MOESM2_ESM.zip › HIVprotI_Source-code/hivprotI/weka-3-6-6/doc/weka/core/OptionHandler.html]

OptionHandler


---


|  |  |  |
| --- | --- | --- |
| **Overview** \| **Package** \| **Class** \| **Tree** \| **Deprecated** \| **Index** \| **Help** \| **Weka's home** | |  |
| **PREV CLASS**   **NEXT CLASS** | **FRAMES**    **NO FRAMES**     **All Classes** |  |
| SUMMARY: NESTED \| FIELD \| CONSTR \| METHOD | DETAIL: FIELD \| CONSTR \| METHOD |  |


---


## weka.core Interface OptionHandler

**All Known Subinterfaces:**: CARuleMiner, DistanceFunction

**All Known Implementing Classes:**: AbstractFileSaver, AbstractStringDistanceFunction, AbstractTimeSeries, AdaBoostM1, Add, AddClassification, AddCluster, AddExpression, AddID, AdditiveRegression, AddNoise, AddValues, ADTree, Agrawal, AllJavadoc, AlphabeticTokenizer, AODE, AODEsr, Apriori, ArffSaver, AttributeSelectedClassifier, AttributeSelection, AveragingResultProducer, Bagging, BallSplitter, BallTree, BallTreeConstructor, BayesianLogisticRegression, BayesNet, BayesNet, BayesNetEstimator, BayesNetGenerator, BestFirst, BFTree, BIFReader, BIRCHCluster, BMAEstimator, BottomUpConstructor, BVDecompose, BVDecomposeSegCVSub, C45Saver, CachedKernel, Center, CfsSubsetEval, ChangeDateFormat, CharacterDelimitedTokenizer, ChebyshevDistance, Check, CheckAssociator, CheckAttributeSelection, CheckClassifier, CheckClusterer, CheckEstimator, CheckGOE, CheckKernel, CheckOptionHandler, CheckScheme, CheckSource, CheckSource, ChiSquaredAttributeEval, CISearchAlgorithm, CitationKNN, ClassAssigner, ClassBalancedND, ClassificationGenerator, ClassificationViaClustering, ClassificationViaRegression, Classifier, ClassifierSplitEvaluator, ClassifierSubsetEval, ClassOrder, CLOPE, ClusterDefinition, ClusterGenerator, ClusterMembership, Cobweb, ComplementNaiveBayes, ConjunctiveRule, Copy, CostSensitiveASEvaluation, CostSensitiveAttributeEval, CostSensitiveClassifier, CostSensitiveClassifierSplitEvaluator, CostSensitiveSubsetEval, CoverTree, CrossValidationResultProducer, CSVLoader, CSVResultListener, CSVSaver, CVParameterSelection, Dagging, DatabaseLoader, DatabaseResultProducer, DatabaseSaver, DataGenerator, DataNearBalancedND, DBScan, DecisionStump, DecisionTable, Decorate, DensityBasedClustererSplitEvaluator, DiscreteEstimator, DiscreteEstimatorBayes, DiscreteEstimatorFullBayes, Discretize, Discretize, DMNBtext, DTNB, EditableBayesNet, EditDistance, EM, END, Estimator, EuclideanDistance, ExhaustiveSearch, Experiment, Expression, FarthestFirst, FilteredAssociator, FilteredAttributeEval, FilteredClassifier, FilteredClusterer, FilteredSubsetEval, FindWithCapabilities, FirstOrder, FPGrowth, FromFile, FT, FTInnerNode, FTLeavesNode, FTNode, FTtree, GainRatioAttributeEval, GaussianProcesses, GeneralizedSequentialPatterns, GeneralRegression, GeneticSearch, GeneticSearch, GeneticSearch, GlobalInfoJavadoc, GlobalScoreSearchAlgorithm, Grading, GreedyStepwise, GridSearch, HierarchicalClusterer, HillClimber, HillClimber, HNB, HyperPipes, IB1, IBk, ICSSearchAlgorithm, Id3, InfoGainAttributeEval, InstanceQuery, InstancesResultListener, InterquartileRange, IsotonicRegression, IteratedSingleClassifierEnhancer, J48, J48graft, Javadoc, JRip, K2, K2, KDTree, KDTreeNodeSplitter, Kernel, KernelEstimator, KernelFilter, KMeansInpiredMethod, KStar, LADTree, LAGDHillClimber, LatentSemanticAnalysis, LBR, LearningRateResultProducer, LeastMedSq, LED24, LibLINEAR, LibSVM, LibSVMSaver, LinearForwardSelection, LinearNNSearch, LinearRegression, ListOptions, LMT, LMTNode, LocalScoreSearchAlgorithm, Logistic, LogisticBase, LogitBoost, LWL, M5Base, M5P, M5Rules, MahalanobisEstimator, Main, MakeDensityBasedClusterer, MakeIndicator, ManhattanDistance, MathExpression, MDD, MedianDistanceFromArbitraryPoint, MedianOfWidestDimension, MedianOfWidestDimension, MergeTwoValues, MetaCost, MexicanHat, MIBoost, MIDD, MiddleOutConstructor, MidPointOfWidestDimension, MIEMDD, MILR, MINND, MIOptimalBall, MIPolyKernel, MIRBFKernel, MISMO, MISVM, MIWrapper, MultiBoostAB, MultiClassClassifier, MultiFilter, MultiInstanceToPropositional, MultilayerPerceptron, MultiNomialBMAEstimator, MultipleClassifiersCombiner, MultiScheme, NaiveBayes, NaiveBayes, NaiveBayesMultinomial, NaiveBayesMultinomialUpdateable, NaiveBayesSimple, NaiveBayesUpdateable, NBTree, ND, NearestNeighbourSearch, NeuralNetwork, NGramTokenizer, NNge, NominalToBinary, NominalToBinary, NominalToString, NormalEstimator, NormalizableDistance, Normalize, Normalize, NormalizedPolyKernel, NumericCleaner, NumericToBinary, NumericToNominal, NumericTransform, OneR, OneRAttributeEval, OPTICS, OptionHandlerJavadoc, OrdinalClassClassifier, PaceRegression, PairedCorrectedTTester, PairedTTester, PART, PartitionedMultiFilter, PKIDiscretize, PLSClassifier, PLSFilter, PMMLClassifier, PointsClosestToFurthestChildren, PoissonEstimator, PolyKernel, PotentialClassIgnorer, PrecomputedKernelMatrixKernel, PreConstructedLinearModel, PredictiveApriori, PrincipalComponents, PrincipalComponents, Prism, PropositionalToMultiInstance, Puk, RacedIncrementalLogitBoost, RaceSearch, RandomCommittee, RandomForest, RandomizableClassifier, RandomizableClusterer, RandomizableDensityBasedClusterer, RandomizableIteratedSingleClassifierEnhancer, RandomizableMultipleClassifiersCombiner, RandomizableSingleClassifierEnhancer, RandomizableSingleClustererEnhancer, Randomize, RandomProjection, RandomRBF, RandomSearch, RandomSplitResultProducer, RandomSubset, RandomSubSpace, RandomTree, Ranker, RankSearch, RBFKernel, RBFNetwork, RDG1, RegOptimizer, Regression, RegressionByDiscretization, RegressionGenerator, RegressionSplitEvaluator, RegSMO, RegSMOImproved, RELAGGS, ReliefFAttributeEval, RemoteExperiment, Remove, RemoveFolds, RemoveFrequentValues, RemoveMisclassified, RemovePercentage, RemoveRange, RemoveType, RemoveUseless, RemoveWithValues, Reorder, RepeatedHillClimber, RepeatedHillClimber, ReplaceMissingValues, REPTree, Resample, Resample, ReservoirSample, Ridor, RotationForest, RuleNode, ScatterSearchV1, SearchAlgorithm, SerializedClassifier, SerializedInstancesSaver, sIB, SimpleBatchFilter, SimpleCart, SimpleEstimator, SimpleFilter, SimpleKMeans, SimpleLinearRegression, SimpleLogistic, SimpleMI, SimpleStreamFilter, SimulatedAnnealing, SimulatedAnnealing, SingleAssociatorEnhancer, SingleClassifierEnhancer, SingleClustererEnhancer, SlidingMidPointOfWidestSide, SMO, SMOreg, SMOTE, SnowballStemmer, SPegasos, SpreadSubsample, Stacking, StackingC, Standardize, StratifiedRemoveFolds, StringKernel, StringToNominal, StringToWordVector, SubsetByExpression, SubsetSizeForwardSelection, SubspaceCluster, SubspaceClusterDefinition, SVMAttributeEval, SVMLightSaver, SwapValues, SymmetricalUncertAttributeEval, TabuSearch, TabuSearch, TAN, TAN, TechnicalInformationHandlerJavadoc, Tertius, TestInstances, TextDirectoryLoader, ThresholdSelector, TimeSeriesDelta, TimeSeriesTranslate, Tokenizer, TopDownConstructor, UserClassifier, VFI, Vote, VotedPerceptron, WAODE, Wavelet, Winnow, WordTokenizer, WrapperSubsetEval, XMeans, XRFFSaver, ZeroR

---

``` public interface OptionHandler ```

Interface to something that understands options.

**Version:**
:   $Revision: 1.8 $

**Author:**
:   Eibe Frank (eibe@cs.waikato.ac.nz), Len Trigg (trigg@cs.waikato.ac.nz)

---

| **Method Summary** | |
| --- | --- |
| `java.lang.String[]` | `getOptions()`             Gets the current option settings for the OptionHandler. |
| `java.util.Enumeration` | `listOptions()`             Returns an enumeration of all the available options.. |
| `void` | `setOptions(java.lang.String[] options)`             Sets the OptionHandler's options using the given list. |

**Method Detail**

### listOptions

```
java.util.Enumeration listOptions()
```

:   Returns an enumeration of all the available options..

    :   **Returns:**: an enumeration of all available options.

---


### setOptions

```
void setOptions(java.lang.String[] options)
                throws java.lang.Exception
```

:   Sets the OptionHandler's options using the given list. All options
    will be set (or reset) during this call (i.e. incremental setting
    of options is not possible).

    :   **Parameters:**: `options` - the list of options as an array of strings **Throws:**: `java.lang.Exception` - if an option is not supported

---


### getOptions

```
java.lang.String[] getOptions()
```

:   Gets the current option settings for the OptionHandler.

    :   **Returns:**: the list of current option settings as an array of strings


---


|  |  |  |
| --- | --- | --- |
| **Overview** \| **Package** \| **Class** \| **Tree** \| **Deprecated** \| **Index** \| **Help** \| **Weka's home** | |  |
| **PREV CLASS**   **NEXT CLASS** | **FRAMES**    **NO FRAMES**     **All Classes** |  |
| SUMMARY: NESTED \| FIELD \| CONSTR \| METHOD | DETAIL: FIELD \| CONSTR \| METHOD |  |


---
